# Psychoanalysis has its place in modern medicine, and neuropsychoanalysis is here to support it

**DOI:** 10.3325/cmj.2015.56.503

**Published:** 2015-10

**Authors:** Anton Glasnović, Goran Babić, Vida Demarin

**Affiliations:** 1 Zagreb University School of Medicine, Zagreb, Croatia *anton.glasnovic@cmj.hr*; 2 Croatian Psychoanalytic Society, Zagreb, Croatia; 3 Croatian Academy of Sciences and Arts, Zagreb, Croatia

Neuropsychoanalysis is an emerging interdisciplinary field of research aimed at applying neuroscientific findings to the psychoanalytic theory and vice versa (1,2). It links two major conceptual frameworks, neuroscience and psychoanalysis. Each field deals with a distinct subject, although both of them are viewed as two separate aspects of the same matter. While neuroscience studies brain biology, its functions and structures that can be objectified, psychoanalysis studies subjective mental processes. However, neuroscience was faced with several questions: Can it, through complex neuronal mechanisms, explain not only workings of the brain, but also workings of the mind? And, how likely is it that reducing the human mind to molecular interactions and neural networks could ever account for complex subjective phenomena, such as sensations, perceptions, feelings, thoughts, and consciousness? It was precisely these questions that prompted the merging of the two disciplines.

The aim of this short essay is to discuss how neuropsychoanalysis originated and to determine its position in modern medicine. The timing when the merging of disciplines took place is also of importance, because accumulation of new knowledge arrived to a critical point where the unification of neuroscience and psychoanalysis seemed inevitable. One of the major figures in neuroscience, Antonio Damasio, has recently implied this potentiality. He challenges the mind-body dualism, introduced by a philosophical view of the mind introduced by the 17th century thinkers, mainly Rene Descartes, and argues that emotions guide (or bias) human behavior and that in decision making individuals use not only cognitive, but emotional processes. According to Damasio, “Descartes’ error” is the dualist separation of mind and body, rationality and emotion. Damasio's theory points out the “decisive function of emotions in navigating the endless stream of life's personal decisions” (3,4).

The other significant dualistic concept named “dual aspect monism” was articulated by Immanuel Kant and Arthur Schopenhauer. During the history of our understanding of the mind, not only philosophers, but also scientists adopted the dualistic view. Jung is one of the prominent figures who emphasized the dual nature of mental and physical states (5). Freud, on the contrary, adopted the monistic approach in his medical research, but due to the stage of technological development of his time, continued to develop only the theory of mind, namely psychoanalytical method. In his writings, he speculated that one day, as technology advances, the paradigm will have to shift back to the monistic approach (6). As neuroscience and psychoanalysis continued to advance separately, there was a challenge of bridging the communication gap between them, mainly due to opposing methodological approaches. This task was not accomplished for most of the century. Driven by advances in research design and methodology and attempts to provide the best practice evidence, neuroscientists and psychologists, similar to experts in other medical fields and disciplines, embraced the evidence-based philosophy. At the same time, psychoanalysis continued to progress in its own setting without implementing research methods used in evidence-based medicine in the everyday practice. This inevitably led to criticism from other fields and again sparked the old fight between the disciplines for dominance over matters of the mind. For example, Edwin Garrigues Boring, an experimental neuropsychologist, argued that “psychoanalysis does not have experiments, and has neither the control nor the ability to distinguish between semantic specification and the facts” (7). Eric Kandel, both neuroscientist and psychiatrist, argued that psychoanalysis was far better at generating ideas then testing them, and therefore did not have the ability to progress in the same way as other areas of research of the mind and medicine in general. He explored the possibilities of biology to refresh the psychoanalytic exploration of the mind (8). On the other hand, psychoanalyst Marshall Edelson holds that biology is irrelevant to psychoanalysis (9), an opinion he shares with many modern psychoanalysts. Despite all these divergent ideas, Mark Solms and several other neuropsychologists who are also psychoanalysts made remarkable progress toward providing a dialogue framework for the two fields, and through their research a new discipline, neuropsychoanalysis, came into existence at the beginning of the third millennium (2,10).

As Freud had foreseen and Kandel asserted, the technology and knowledge had advanced to a point where all prerequisites were met for a dialogue of the two disciplines through a monistic approach. The development of new clinical and laboratory methods such as electroencephalography, evoked potentials, genetic methods, and imaging methods made it possible to bring neuroscience and psychoanalysis closer together than ever before. One of the most interesting examples of such cooperation is the explanation of hemispacial neglect of the left side of the body after brain damage. The left sided hemispacial neglect was traditionally thought to be the consequence of the physical damage of the contralateral hemisphere. However, this view was challenged after detailed observation of several such patients in a psychoanalytic situation. The results of these observations imply a different explanatory mechanism, namely that the phenomenon might be a consequence of various complex defense mechanisms of the unconscious (11,12). Another interesting breakthrough in this field came from the research of the dreaming process. According to findings from studies on this subject matter, the thought process is constantly activated during the awake state, as well as during sleep, and the dreams, more or less structured, can appear during every phase of the sleeping state, not only as we usually thought, during the rapid eye movement phase. The most important dream generator is a sufficiently intense arousal stimulus that initiates the dreaming process by deactivating the motor and premotor frontal cortex. Cortical deactivation releases the mesocortical and mesolimbic “seeking systems,” and activates the associative cortex of the occipito-temporo-parietal junction to form an illusion, which we call a dream (12). All of this is in absolute concordance with Freud’s idea that the dream protects the subject from waking, and that the thought process is continuous during sleep, but that it is converted into dreaming under various stimuli. The dreaming process is not under control of the super-ego, which is more or less the case in the awake state (13,14).

Still, some scientists mistrust the idea of neuropsychoanalysis, claiming that neither of the fields will have much to gain from this conjunction. One of the skeptics is the cognitive neuroscientist Marck Ramus, who argues “that the science of the mind already exists, and that is psychology.” Psychology already cooperates with neuroscience, therefore he sees neuropsychoanalysis as “nothing more than an attempt to rehabilitate psychoanalysis by giving it a fashionable prefix and by attributing it the merits of other disciplines.” Ramus goes one step further and warns about the perils of psychoanalytic treatment in certain cases. He gives the example of unsupported psychoanalytical approach in autism, claiming that “psychoanalysis rejects international classification of mental disorders in favor of their own idiosyncratic ones.” By practicing analytical forms of psychotherapy, whose efficacy is not supported by any empirical evidence, psychoanalysts, in his opinion, delay the diagnosis of autism and subsequent educational intervention (15).

So, after a glimpse into some of the opposing arguments, can we predict the future of neuropsychoanalysis? Or maybe psychoanalysis as such? We think that, in order to see things more clearly, a change in perspective is paramount. The subject of psychoanalysis is not the objective reality of an individual, whose features and attributes can be counted and statistically analyzed like in somatic diseases, where one can measure, for example, blood glucose levels or prove the existence of objective markers of hip arthrosis or carotid artery stenosis. Psychoanalysis deals with the feelings that patients have about themselves and the surrounding world. It also deals with the subjective matter of things, whereas neuroscience deals with the objective matter of things. This notion makes psychoanalysis also an art form, and art cannot be quantified like blood pressure or neurotransmitter concentration in the synaptic cleft (16,17). However, the practitioner’s skill and technique can be evaluated, as well as the level of inherent meaning of the approach, and eventually its intent to fulfill its purpose, that is, to help the patient. When trying to account for the complexity of the individual psyche, psychology, as a “science of the mind“ (15) due to its reductionist concepts and clinical methodology, faces somewhat similar problems. Indeed the psyche is not equal to cognition or even less to consciousness, but is something much wider. In order to comprehend it, and to help individuals suffering from mental disorders we have to leave our prejudice behind and move away from the narcissistic phase, but this time armed with new technologies and new perspectives. Although the research setting of neuroscience is the lab bench, and the setting of psychoanalysis is the couch, new interesting insights into our mental functioning arrive daily from both of the disciplines. This prompts us to move from brain to bedside, and vice versa in order to gain a deeper knowledge about ourselves.
